# Trends in Amplitude-Integrated Electroencephalography in the Smallest Preterm Neonates

**DOI:** 10.3390/children11050566

**Published:** 2024-05-08

**Authors:** Kristina Štuikienė, Elke Griesmaier, Ilona Aldakauskienė, Regina Vidmantė, Kastytis Šmigelskas, Rasa Tamelienė

**Affiliations:** 1 Department of Neonatology, Lithuanian University of Health Sciences, 44307 Kaunas, Lithuania; 2 Department of Pediatrics II, Medical University of Innsbruck, 6020 Innsbruck, Austria; 3 Health Research Institute, Faculty of Public Health, Lithuanian University of Health Sciences, 44307 Kaunas, Lithuania

**Keywords:** amplitude-integrated EEG, premature infant, neonatal brain function, Burdjalov score

## Abstract

Background. Amplitude-integrated electroencephalography is increasingly used for the neuromonitoring of premature infants. However, it is still not clear how bioelectrical activity changes in the smallest gestational age newborns. The aim of our study was to evaluate the bioelectrical activity of amplitude-integrated electroencephalograms in premature newborns of different gestational age to assess how gestational age and postnatal age influence patterns of amplitude-integrated electroencephalograms and to test the hypothesis of whether the bioelectrical activity of the brain matures faster after the birth of premature newborns than in utero. Methods. We prospectively included infants born before 32 weeks of gestational age between June 2020 and July 2022. Serial recordings of amplitude-integrated electroencephalograms were performed at three time points of age (days 1–3, 13–15, and 27–29). Recordings were analyzed for background patterns, the onset and appearance of cyclicity, and lower amplitude border and bandwidth, which were used to derive a composite Burdjalov score. Results. In total, 140 premature neonates were included in the study, and 112 of them completed the study. The median gestational age of the newborns enrolled in the study was 29 (27–30) weeks, and the mean weight was 1206 (350) g. Burdjalov scores increased with increasing gestational age. Higher scores were observed in every dimension of the amplitude-integrated electroencephalograms for newborns of lower gestational age when compared to newborns of higher gestational age of the same postmenstrual age. There was a significant correlation between gestational age and parameters of amplitude-integrated electroencephalograms at all time points. Conclusions. A higher gestational age has a positive effect on the bioelectrical activity of amplitude-integrated electroencephalograms. Increasing postnatal age affected amplitude-integrated electroencephalograms more than gestational age. Our hypothesis that the bioelectrical activity of the brain matures faster for premature newborns after birth than in the womb was confirmed.

## 1. Introduction

Advancements in modern perinatal medicine and in the intensive care of preterm infants have improved their survival rates [1,2,3,4]. However, despite decreasing mortality, prematurity remains the leading cause of early death in premature infants. Such babies are most affected by dangerous complications, which occur during the neonatal period and often damage the brain [4,5]. As a result, the quality of life of premature newborns with perinatal brain damage has become a focus of attention [1]. Over the years, amplitude-integrated electroencephalography (aEEG) has been increasingly integrated in neonatology as a method for the early detection of brain damage. Limited-channel aEEG monitoring provides a simplified form of EEG monitoring that can be performed over prolonged periods of time in the Neonatal Intensive Care Unit (NICU). In recent decades, aEEG monitoring has become especially important for premature newborns. However, the role of aEEG monitoring in premature infants remains less clear [1,6].

The interpretation of an aEEG tracing includes three categories: the classification of the background pattern, the identification of cyclicity (sleep–wake cycling), and seizures [1,7,8,9].

Two major factors in the classification of aEEG traces are voltage and pattern. Voltages in the trace are classified as normal, abnormal, and low [9]. The background patterns are classified according to the upper and lower limit values [7,9,10]: continuous normal voltage activity with a lower amplitude > 5 µV and a maximum amplitude > 10–25 µV; discontinuous normal voltage activity with a minimum amplitude < 5 µV and a maximum amplitude > 10 µV; burst–suppression—discontinuous activity with a minimum amplitude without variability at 0–1 µV and bursts with an upper amplitude > 25 µV; continuous low voltage—continuous activity of very low amplitude, where when the lower and upper limits are <5 µV, there may be some variables; flat (isoelectric line)—mainly inactive background < 5 µV, without variables.

Sleep–wake cycling in aEEG is characterized by smooth cyclic changes, mainly of the amplitude of the lower border. It can be seen in healthy term babies. The developed sleep–wake cycle seems to curl up and down with narrowing and expanding traces. Periods with broader parts of aEEG represent more discontinuous activity during quiet sleep, and the narrower parts correspond to more continuous background activity during wakefulness or active sleep [7,8,9]. The immature sleep–wake cycle is defined as not fully developed cyclic variation in the lower border amplitude when compared with full-term infant aEEG data. The absence (no sleep–wake cycle) of sleep–wake cycling is when there are no sinusoidal variations in the aEEG background. This can happen in very sick full-term neonates. Also, immature sleep–wake cycling or its absence can indicate the presence of an immature brain, and this can be seen in very premature infants [7,9].

Seizures are described as a sudden increase in lower and upper amplitude, often followed by a postictal phase of decreased amplitude [7,10].

Normal aEEG of a healthy full-term infant is characterized by continuous normal voltage, with clear sleep–wake cycling and without seizure activity. Other types of background activity of aEEG can only be seen in sick full-term newborns, and it depends on the severity of the condition of the baby [7,8,9].

Normal background aEEG in very preterm infants is discontinuous and characterized by high-voltage bursts and low-voltage interburst intervals [11]. Background activity changes with increasing gestational age (GA) and becomes more continuous [9,12].

The sleep–wake pattern is poorly developed in preterm neonates [6]. In many extremely preterm neonates, there are no waves in the lower margin of the aEEG in the first days after birth. As maturity increases, a certain but not fully developed cyclic variation of the lower border amplitude can be distinguished, but it is not developed compared to the normative data of full-term neonates [7]. The beginning of sleep–wake cycles can be observed in infants who are in good clinical condition even at 23 weeks of gestation [13], but mostly at 25–26 weeks of gestation [1]. Clear sleep–wake cycles can be observed from 30 to 31 weeks [14].

There are conditions that can affect aEEG changes in newborns, such as a certain conditions during transition, intraventricular hemorrhages, sedative and analgesic medications, and acute periods of some diseases [13,15,16,17,18,19,20,21,22,23]. Therefore, it is very important to take this into account when evaluating physiological changes in aEEG.

Over the past two decades, several studies have been conducted on the effects of maturation in the aEEG monitoring of preterm infants and how postnatal age influences brain maturation [13,24,25,26,27,28]. However, most authors state that additional research is needed.

The aim of our study was to evaluate aEEG bioelectrical activity in premature newborns of different gestational ages and to assess how GA and postnatal age (PNA) influence the aEEG to test the hypothesis of whether the bioelectrical activity of the brain matures faster after the birth of premature newborns than in utero.

## 2. Materials and Methods

This prospective study was conducted at the Department of Neonatology of the Hospital of Lithuanian University of Health Sciences (HLUHS) Kaunas Clinics.

The protocol was approved by the Kaunas Regional Biomedical Research Ethics Committee, and permit No. BE-2-80 was issued on 3 October 2019. The study was registered in the ENCPP database (EUPAS35709).

### 2.1. Study Population

The study was conducted from 1 June 2020 to 30 July 2022. Preterm infants born and treated in the Department of Neonatology of the Hospital of Lithuanian University of Health Sciences Kaunas Clinics were included in this study.

Subject inclusion criteria were as follows: neonates born between 22 + 0 and 31 + 6 weeks of gestation and written consent to participate in the study obtained from both parents. We excluded infants who had multiple developmental defects and chromosomal anomalies, metabolic diseases caused by a genetic disorder, intraventricular hemorrhage (IVH) grade III–IV (intraparenchymal), cystic periventricular leukomalacia (PVL), progressive posthemorrhagic hydrocephalus, or meningitis and those who did not complete the study due to death.

### 2.2. aEEG Monitoring

Serial aEEG recordings were performed during the first 4 weeks of life. An amplitude-integrated electroencephalogram was recorded for at least 6 h at three time points: days 1–3 (first electroencephalogram), 13–15 (second electroencephalogram), and 27–29 (third electroencephalogram) of PNA.

The subjects were divided into four groups according to gestational age: group 1—23–25 weeks, group 2—26–27 weeks, group 3—28–29 weeks, and group 4—30–31 weeks.

Postnatal age was defined as chronological age (time elapsed from birth). Postmenstrual age equals gestational age plus postnatal age.

The Olympic Brainz Monitor (CE0086, BrainZ instruments, Natus Medical Incorporated, Middleton, WI, USA), a two-channel bedside aEEG monitor that displays raw and amplitude-compressed recordings for each hemisphere, was employed in this study. For each recording, a qualified and experienced researcher (certificate Nos. 6383-9531-3706 and 2179-2204-3706) placed cup electrodes at the C3, P3, C4, and P4 regions. We analyzed (P3 and P4) cross-cerebral aEEG recordings.

Data of continuous cerebral activity were obtained and stored in the Olympic Brainz Monitor then evaluated using Olympic Brainz Viewer software (version number OBM00001). The recording quality was monitored by continuous measurement of electrode impedance. Traces with an impedance >10 kOhm or with obvious or marked artifacts were excluded from the analysis.

### 2.3. aEEG Analysis

From each recording, the most stable uninterrupted period of at least 6 hs’ duration was chosen for analysis. The aEEG tracings were assessed according to the proposed Burdjalov scoring system, which is used to assess brain maturation in newborns [11]. The following 4 components were evaluated: the background pattern, onset and appearance of cyclicity, lower amplitude border, and bandwidth span (bandwidth reflects a combination of the voltage span (peak-to-trough) of the tracing and the magnitude of the aEEG depression (amplitude of the lower border); the span is the narrowest part between the upper and lower voltage margins of the tracing) (Table 1). Each variable was scored, and the individual scores were summed to determine the total score for each recording. The maximum possible total score was 13. There was no sedation, acute stage of necrotizing enterocolitis (NEC), or sepsis during the aEEG recordings. All recordings were analyzed by one aEEG analyst.

#### Cranial Ultrasound

Cerebral ultrasound scans were performed (as per the study protocol, performed with the device LOGIQ S8, GE Ultrasound Korea, Seongnam-Si, Republic of Korea) at five time points: the third day and the first, second, third, and fourth week of life. Ultrasound scans were performed and assessed by an experienced radiologist, a qualified specialist in ultrasound examination. IVH and PVL were classified according to Burstein et al. [29] and de Vries et al. [30], respectively.

### 2.4. Collection of Clinical Data

The main characteristics and clinical data of the subjects were obtained from medical documentation. Premature rupture of membranes (PROM) was defined as the rupture of membranes occurring more than 24 h before delivery, a diagnosis of early-onset (<72 h of birth) or late-onset (>72 h) sepsis required signs of generalized infection confirmed by laboratory tests, necrotizing enterocolitis was defined according to Bell’s criteria [31], hemodynamic significance of patent ductus arteriosus (PDA) was confirmed by echocardiography, and bronchopulmonary dysplasia (BPD) was defined by a requirement of oxygen supplementation at the 28th day of life [32]. Gestational age (completed weeks) was defined as the time elapsed from the first day of the last menstrual period to the day of delivery.

Cerebral hemorrhage was categorized into grades: I (isolated germinal matrix hemorrhage), II (intraventricular extension of hemorrhage with normal ventricular size), III (intraventricular hemorrhage with dilated ventricles), and IV (grade III plus extension of germinal matrix hemorrhage into adjacent brain parenchyma) [29]. Periventricular leukomalacia was diagnosed by the presence of localized or extensive echo-lucent areas in the periventricular area [30]. Progressive posthemorrhagic hydrocephalus was defined as hydrocephalus after intraventricular hemorrhage in preterm infants.

### 2.5. Statistical Analysis

The data analysis was performed using the SPSS software version 28.0 (IBM Corp., Released 2021, IBM SPSS Statistics for Windows, Version 28.0., Armonk, NY, USA: IBM Corp). The significance level was set at *p* < 0.05. The descriptive statistics were calculated using mean and median for central tendency as well as standard deviation and interquartile range for the spread of the continuous indicators. The group variables were described in absolute prevalences (n) and percentages (%).

The relationships between ordinal and continuous variables were assessed using the Spearman’s rank correlation (rho). The statistical significance was set at *p* < 0.05.

## 3. Results

### 3.1. Study Population

**Figure 1:**
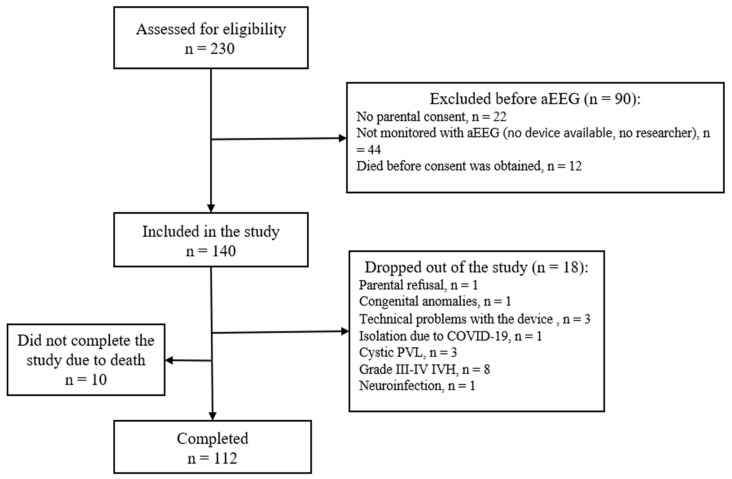
Flow diagram of the study subjects’ inclusion and exclusion.

During the whole study period, 230 neonates eligible for the study were born and treated in the Department of Neonatology of the HLUHS Kaunas Clinics. A total of 140 newborns were included in the study, and 112 of them completed it; 10 neonates did not complete the study due to death (22 weeks—1; 23 weeks—2; 24 weeks—1; 25 weeks—4; 26 weeks—1; 27 weeks—1). A flow diagram of the study subjects’ inclusion and exclusion is shown in Figure 1.

The median gestational age of the newborns included in the study was 29 (27–30) weeks, and the mean weight was 1206 (350) g. All main initial characteristics of the subjects are provided in Table 2.

### 3.2. aEEG Analysis

The mean (SD) start time of the first aEEG recording was 41 (16) h after birth.

### 3.3. Continuity

**Figure 2:**
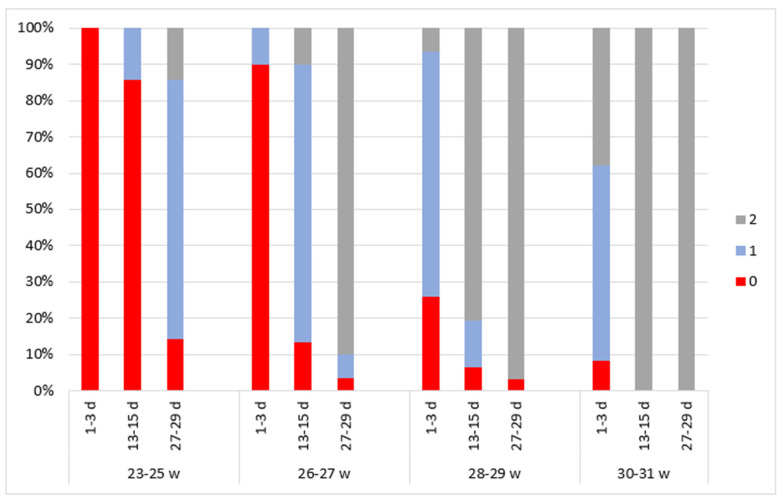
The distribution of continuity Burdjalov scores in four groups according to gestational and postnatal age. The graph is divided into four parts according to gestational age groups. Different aEEG assessment times are indicated on the *x*-axis. The aEEG score for each criterion is colored.

During the analysis of aEEG bioelectrical activity and continuity (Figure 2), a discontinuous pattern (score of 0) was observed in every subject of the 23–25 week GA group immediately after birth. As the newborns became older, their bioelectrical activity changed, and at a PNA of 4 weeks the continuity was evaluated at a score of 2 for 14.3% of the subjects and at a score of 1 for 71.4%.

Similar outcomes were identified in the 26–27 week GA group. Immediately after birth, a discontinuous pattern (score of 0) was observed in 90% of the subjects. At a PNA of 2 weeks, a continuous pattern (score of 2) was detected in 10% of the neonates and a somewhat continuous pattern (score of 1) in 76.7%. At a PNA of 4 weeks, a definite continuous pattern (score of 2) was identified in 90% of the subjects.

We observed better bioelectrical activity immediately after birth in groups of higher gestational age. In the 28–29 week GA group, continuity was evaluated at a score of 2 in 6.5% of the subjects immediately after birth and at a score of 1 in 67.7%. A score of 2 was detected in 80.6% of the subjects at a PNA of 2 weeks and in 96.8% at a PNA of 4 weeks.

In the 30–31 week GA group, a score of 2 was observed in 37.8% of the subjects immediately after birth and in 100% at a PNA of 2 and 4 weeks.

### 3.4. Cyclicity

**Figure 3:**
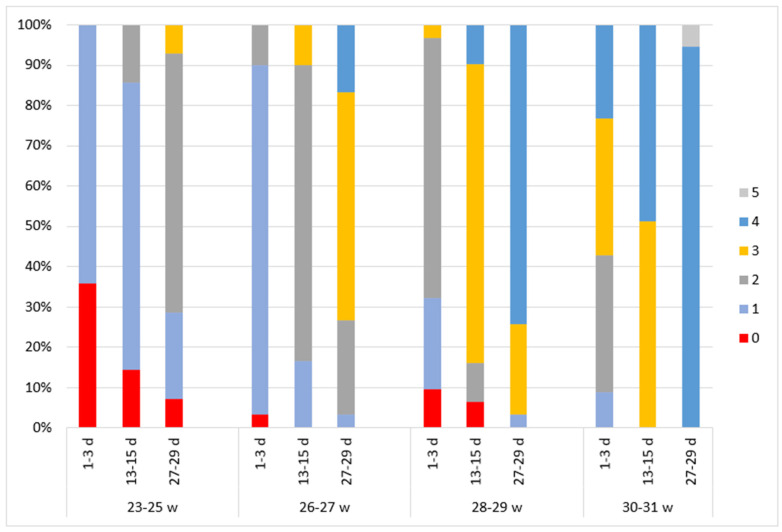
The distribution of cycling Burdjalov scores in four groups according to gestational and postnatal age. The graph is divided into four parts according to gestational age groups. Different aEEG assessment times are indicated on the *x*-axis. The aEEG score for each criterion is colored.

In the 23–25 week GA group, beginning cyclicity (score of 1) was detected in 64.3% of the subjects immediately after birth (Figure 3). At a PNA of 2 weeks, this was observed in 71.4% of the newborns, and cycling was evaluated at a score of 2 for 14.3% of the subjects. At a PNA of 4 weeks, developing cyclicity (score of 2) was identified in 64.3% of the subjects and definite cycling (score 3) in 7.1%.

In the 26–27 week GA group, beginning cyclicity (score of 1) was observed in 86.7% of the newborns immediately after birth. Developing cyclicity (score of 2) was detected in 73.3% of the subjects at a PNA of 2 weeks, and mature cycling was predominant in the aEEG at a PNA of 4 weeks: definite, noninterrupted cycling (score of 4) in 16.7% of the subjects and cyclicity with interruptions (score of 3) in 56.7%.

Immature cyclicity (score of 2) was predominant in the 28–29 week GA group (64.5%) of the subjects immediately after birth. As the newborns became older, most of them developed mature cyclicity, and at a PNA of 4 weeks, 74.2% of them were evaluated at a score of 4 and 22.6% at a score of 3.

Some of the subjects (51.4%) in the 30–31 week GA group already had definite cyclicity (score of 3) immediately after birth. Definite, noninterrupted cycling (score of 4) was observed in 48.6% of the subjects at a PNA of 2 weeks and in 94.6% at a PNA of 4 weeks. A small part of the group (5.4%) was assessed to have regular mature cycling (score 5).

### 3.5. Amplitude of the Lower Border

**Figure 4:**
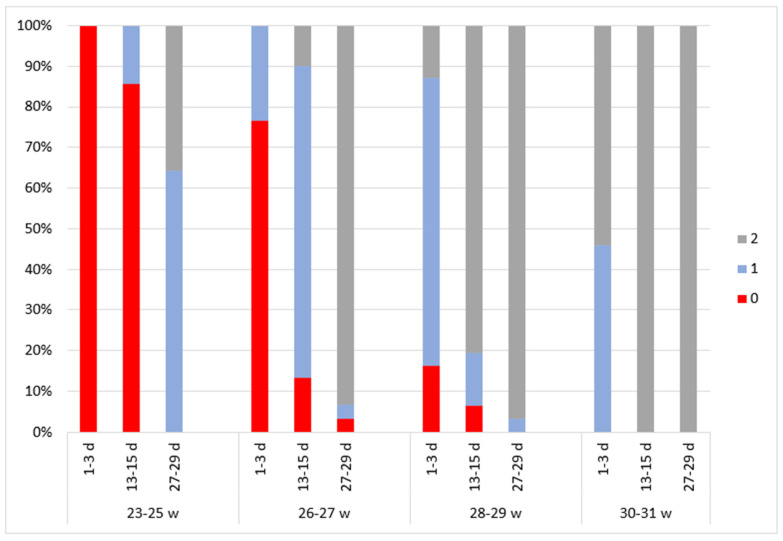
The distribution of amplitude of the lower border Burdjalov scores in four groups according to gestational and postnatal age. The graph is divided into four parts according to gestational age groups. Different aEEG assessment times are indicated on the *x*-axis. The aEEG score for each criterion is colored.

When analyzing the amplitude of the lower border (ALB) (Figure 4), every subject of the 23–25 week GA group had a severely depressed amplitude of the lower border (score of 0) immediately after birth. The ALB was evaluated at a score of 1 for 14.3% of the subjects at a PNA of 2 weeks and at a score of 2 for 35.7% at a PNA of 4 weeks.

Similar results were obtained in the 26–27 week GA group immediately after birth, as the ALB for 76.7% of the subjects was evaluated at a score of 0. An elevation of traces (score of 2) was observed in 10% of the subjects at a PNA of 2 weeks and in 93.3% at a PNA of 4 weeks.

In the 28–29 week GA group, elevated traces (score of 2) were identified in 12.9% of the subjects immediately after birth, in 80.6% at a PNA of 2 weeks, and in most subjects (96.8%) at a PNA of 4 weeks.

Of the subjects in the 30–31 week GA group, 54.1% had elevated traces (score of 2) immediately after birth. This was observed in 100% of the participants at a PNA of 2 and 4 weeks.

### 3.6. Bandwidth Span and Amplitude of the Lower Border

**Figure 5:**
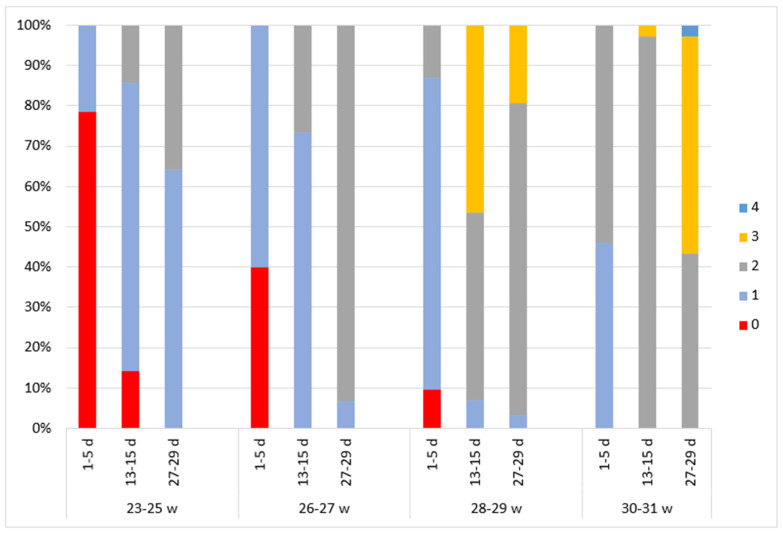
The distribution of bandwidth span and amplitude of the lower border Burdjalov scores in four groups according to gestational and postnatal age. The graph is divided into four parts according to gestational age groups. Different aEEG assessment times are indicated on the *x*-axis. The aEEG score for each criterion is colored.

When analyzing the bandwidth span (BS) and amplitude of the lower border (Figure 5), 21.4% of the subjects in the 23–25 week GA group were evaluated at a score of 1 immediately after birth. The BS was assessed at a score of 2 for 14.3% of the participants at a PNA of 2 weeks and for 35.7% at a PNA of 4 weeks.

Of the subjects in the 26–27 week GA group, 60% received a BS evaluation score of 1 immediately after birth. The BS was assessed at a score of 2 for 26.7% of the participants at a PNA of 2 weeks and for 93.3% at a PNA of 4 weeks.

The results were better immediately after birth in groups of higher gestational age. In the 28–29 week GA group, 12.9% of the subjects received a BS evaluation score of 2; 87.1% of the newborns were observed to have a BS at a score of 2 at a PNA of 2 weeks, and 19.4% of the subjects had a maturing BS (score of 3) at a PNA of 4 weeks.

Of the subjects in the 30–31 week GA group, 54.1% had a BS evaluation score of 2 immediately after birth. A maturing BS (score of 3) was identified in 2.7% of the subjects at a PNA of 2 weeks and in 54.1% at a PNA of 4 weeks. A small part of the group (2.7%) had a mature BS (score 4) at a PNA of 4 weeks.

**Figure 6:**
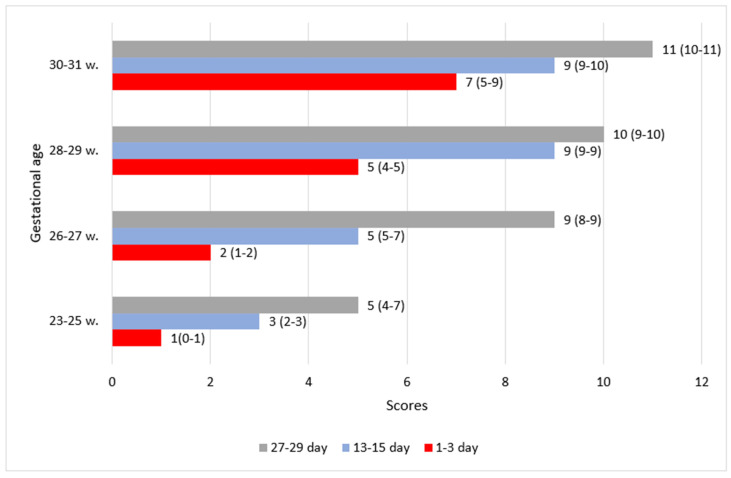
The distribution of total Burdjalov scores in the four groups according to gestational and postnatal age. The graph is divided into four parts according to gestational age groups on the *y*-axis. The total Burdjalov scores of the aEEG evaluations are indicated on the *x*-axis. The different time points of the aEEG assessments are colored. The Burdjalov scores are presented in median (IQR).

The total score increased with GA (Figure 6). The 23–25 and 26–27 gestational groups started with scores of 1 and 2, while the other two groups (28–29 and 30–31 weeks) started with scores of 5 and 7. In all groups, the total score increased with PNA. The highest score of 11 was observed in the group of neonates of 30–31 weeks of a GA of 4 weeks.

In newborns of lower gestational age, higher scores were observed in every dimension of the aEEG assessment compared to higher GA newborns of the same PMA (Figure 2, Figure 3, Figure 4, Figure 5 and Figure 6).

There was a significant correlation between GA and aEEG scores in all dimensions at all time points (r = 0.45–0.84; *p* < 0.01) (Table 3).

## 4. Discussion

In this study, changes in aEEG trends during the first 4 weeks of life in extremely preterm infants born between 23 and 31 weeks of gestational age are described. The data of individual aEEG parameters according to gestational age groups and their evolution with increasing PNA are presented. Our results demonstrate that the maturation of the aEEG tracing depends on both GA and PNA. There is a strong correlation between GA and aEEG parameters at all time points. Our study shows that newborns of lower gestational age were evaluated with higher scores in every dimension of the aEEG than the newborns of higher GA of the same PMA.

The main idea of the study was to investigate physiological changes in aEEG assessments in the smallest preterm neonates. Therefore, in our study, we excluded neonates with IVH grade III–IV, cystic PVL, progressive posthemorrhagic hydrocephalus, and neuroinfection. There was no sedation or acute period of sepsis or NEC during the aEEG recordings. According to clinical studies [13,16,17,18,19,20,21,22,23], the listed conditions can affect aEEG background patterns. In our study, the first aEEG measurement of the subjects was recorded approximately after the first day of life. We can state that transition after birth and perinatal factors did not have much effect on the infants’ amplitude-integrated electroencephalograms [13,25,33].

Despite the immaturity of the newborns and the influence of transition, the presence of aEEG continuity was detected in the group of the smallest infants of 23–25 weeks of gestational age immediately after birth. Similar results were reported by Hayakawa et al. [12].

However, a discontinuous pattern, severely depressed traces, and a very immature BS were predominant in the 23–27 week GA group immediately after birth, whereas the aEEG result was more mature in the 28–31 week GA group from the very first days, showing predominant continuity, an elevated ALB, and an immature BS. These results are not surprising, as maturity differs greatly between 22–27 and 28–31 week GA newborns in other systems as well. A study by Burdjalov et al., who used the same aEEG scoring system [11], as well as Han et al. [34] and Reda et al. [35], showed similar results. However, all mentioned studies had different subject inclusion criteria and aEEG monitoring periods. The research of Niemarkt et al. showed that with advancing GA, quantitative aEEG parameters such as ‘lower margin amplitude’ increased and ‘discontinuous pattern’ decreased, and there was a significant correlation between these parameters and GA [36].

The evaluation of cycling in different GA groups helped to determine a clear pattern. With higher GA, a clearer and more mature cyclicity of bioelectrical activity was observed immediately after birth. It may be concluded that sleep–wake cycles are the best representations of GA and maturity of the brain [37]. A similar tendency was discovered in a study conducted by Burdjalov et al. [11]. Beginning cyclicity was observed in the least-developed newborn group (23–25 week GA), with similar results obtained by Olischar et al. [38] and Deshpande et al. [13].

During the analysis of the changes in bioelectrical activity cycling according to PMA in different GA groups, definite, noninterrupted cyclicity was detected from a PMA of 32 to 33 weeks, and completely regular and mature cycling was identified for some of the subjects at a PMA of 34–35 weeks. However, in research conducted by Han et al. [34], completely regular and mature cycling was not observed until a PMA of 36 weeks.

After assessing how postnatal age impacts the aEEG results in our study, we determined that as the newborns became older, their aEEG bioelectrical activity became more mature. To summarize the results, it may be concluded that postnatal age had a greater impact on the aEEG results than increasing GA, possibly due to a multitude of external auditory and pain stimuli: different diagnostic and treatment procedures, surrounding noise from various devices and personnel, as well as light stimuli, which cause as much stress. Our results are confirmed by the research of Klebermass et al., which determined that both GA and PNA have a significant influence on the occurrence of the discontinuous low-voltage pattern and the continuous pattern [28]. Griesmaier et al. found that PNA had a greater influence on aEEG parameters than GA [39].

We found out that newborns of lower GA were evaluated with a higher score than infants of greater GA at the same PMA.

We monitored the newborns only until a PNA of 4 weeks, and thus we were unable to compare newborns of the smallest GA with newborns of the 30–31 week GA group at the same PMA. The hypothesis we raised about bioelectrical activity of the brain in premature newborns maturing faster that in the womb was confirmed. This is corroborated by research conducted by Soubasi et al. [26] and Sisman et al. [24], although the second researcher’s aEEG assessment methodology was different.

### 4.1. Strengths

The sample of our study included newborns of the lowest gestational age (from 23 weeks). A strength of this study is the relatively large sample size.

### 4.2. Limitations

The subject groups of different GA were of different sizes, with the number of participants being smaller in the groups of lower GA. As such, the obtained data may have been influenced by random factors.

The subjects were monitored for only 4 weeks after birth independently of GA; thus, we cannot compare the subjects of higher gestation at 4 weeks of age with those of lower gestation at the same PMA.

The amplitude-integrated electroencephalograms were assessed by only one qualified specialist.

## 5. Conclusions

A higher gestational age has a positive effect on aEEG bioelectrical activity. Cyclicity is the best indicator of gestational age. Increasing age after birth was a more important factor for the aEEG measurements than gestational age. Our hypothesis that bioelectrical activity of the brain matures faster for premature newborns after birth than in the womb was confirmed. This conclusion was met as newborns of smaller gestational age of the same PMA but of greater postnatal age were evaluated with higher Burdjalov scores than newborns of higher gestational age.

## Figures and Tables

**Table 1:** Burdjalov scoring system of amplitude-integrated electroencephalography [11].

Score	Continuity	Cycling	Amplitude of Lower Border	Bandwidth Span and Amplitude of Lower Border
0	Discontinuous	None	Severely depressed (<3 μV)	Very depressed: low span (≤15 μV) and low voltage (5 μV)
1	Somewhat continuous	Waves first appear	Somewhat depressed (3–5 μV)	Very immature: high (>20 μV) or moderate (15–20 μV) span and low voltage (5 μV)
2	Continuous	Not definite, somewhat cycling	Elevated (>5 μV)	Immature: high span (>20 μV) and high voltage (>5 μV)
3		Definite cycling, but interrupted		Maturing: moderate span (15–20 μV) and high voltage (>5 μV)
4		Definite cycling, noninterrupted		Mature: low span (<15 μV) and high voltage (>5 μV)
5		Regular and mature cycling		

**Table 2:** The main characteristics of the study subjects.

Variable	n = 112
Cesarean section, n (%)	52 (46)
PROM, n (%)	45 (40)
Gestational age (weeks), median (IQR)	29 (27–30)
Gestation age, n (%):	
23 weeks	2 (2)
24 weeks	7 (6)
25 weeks	5 (5)
26 weeks	10 (9)
27 weeks	20 (18)
28 weeks	9 (8)
29 weeks	22 (20)
30 weeks	19 (17)
31 weeks	18 (16)
Birth weight (g), mean (SD)	1206 (350)
Gender, n (%):	
male	62 (55)
female	50 (45)
APGAR score, median (IQR)	
1 min	7 (6–8)
5 min	8 (7–9)
Morbidity	
Late-onset sepsis, n (%)	25 (22)
Necrotizing enterocolitis, n (%)	27 (24)
Patent ductus arteriosus, n (%)	38 (34)
Bronchopulmonary dysplasia, n (%)	23 (21)

PROM—premature rupture of membranes.

**Table 3:** Spearman correlation coefficients between aEEG measurements and gestational age.

Burdjalov Scores	Gestational Age
PNA 1–3 days:	
continuity	0.77 *
cycling	0.71 *
amplitude of lower border	0.81 *
bandwidth span and amplitude of lower border	0.72 *
total score	0.81 *
PNA 13–15 days:	
continuity	0.83 *
cycling	0.84 *
amplitude of lower border	0.72 *
bandwidth span and amplitude of lower border	0.74 *
total score	0.84 *
PNA 27–29 days:	
continuity	0.53 *
cycling	0.80 *
amplitude of lower border	0.45 *
bandwidth span and amplitude of lower border	0.67 *
total score	0.83 *

PNA—postnatal age. * *p* < 0.01.

## Data Availability

The data presented in this study are available on request from the corresponding author. The data are not publicly available due to subject confidentiality.

## Abbreviations

AEEG	amplitude-integrated electroencephalography.
BPD	bronchopulmonary dysplasia.
GA	gestational age.
IVH	intraventricular hemorrhage.
NEC	necrotizing enterocolitis.
NICU	Neonatal Intensive Care Unit.
PDA	patent ductus arteriosus.
PMA	postmenstrual age.
PNA	postnatal age.
PROM	premature rupture of membranes.
PVL	periventricular leukomalacia.
